# *Cistanche tubulosa* phenylethanoid glycosides induce apoptosis in H22 hepatocellular carcinoma cells through both extrinsic and intrinsic signaling pathways

**DOI:** 10.1186/s12906-018-2201-1

**Published:** 2018-10-12

**Authors:** Pengfei Yuan, Jinyu Li, Adila Aipire, Yi Yang, Lijie Xia, Xinhui Wang, Yijie Li, Jinyao Li

**Affiliations:** 1 Xinjiang Key Laboratory of Biological Resources and Genetic Engineering, College of Life Science and Technology, Xinjiang University, 666 Shengli Road, Urumqi, Xinjiang, 830046 China; 2 College of Life Science, Xinjiang Normal University, 102 Xinyi Road, Urumqi, 830054 Xinjiang China; 3 Affiliated Tumor Hospital of Xinjiang Medical University, Urumqi, 830011 China

**Keywords:** *Cistanche tubulosa*, Phenylethanoid glycosides, Apoptosis, Signaling pathway, Tumor mouse model

## Abstract

**Background:**

*Cistanche tubulosa* (Schenk) R. Wight is a traditional Chinese medicine that parasitizes the roots of the Tamarix plant and has been used to treat male impotence, sterility, body weakness, and as a tonic. However, its antitumor effect on hepatocellular carcinoma is still elusive. Here, we investigated the antitumor effect of *C. tubulosa* phenylethanoid glycosides (CTPG) on H22 hepatocellular carcinoma cells both in vitro and in vivo and its mechanisms.

**Methods:**

The morphology, viability, apoptosis, cell cycle and mitochondrial membrane potential (Δψm) of H22 cells were analyzed by inverted microscopy, MTT assay and flow cytometry, respectively. The expression and activation of proteins in apoptosis pathway were detected by Western blot. The in vivo antitumor effect was evaluated in tumor mouse model established using male Kunming mice.

**Results:**

CTPG treatment significantly suppressed H22 cell growth in a dose- and time-dependent manner, which was correlated with the increased apoptosis and cell cycle arrest at G0/G1 and G2/M phases. Moreover, the chromosomal condensation was observed in CTPG-treated H22 cells. CTPG treatment significantly increased Bax/Bcl-2 ratio, reduced Δψm and enhanced the release of cytochrome c. The levels of cleaved caspase-8 and caspase-9 in both extrinsic and intrinsic signaling pathways were significantly increased that sequentially activated caspase-7 and -3 to cleave PARP. Finally, CTPG inhibited the growth of H22 cells in mice and improved the survival rate of tumor mice.

**Conclusions:**

These results suggested that CTPG suppressed H22 cell growth through both extrinsic and intrinsic apoptosis pathways.

## Background

Liver cancer ranked sixth for cancer incidence and fourth for cancer deaths worldwide. Moreover, it ranked fourth for cancer incidence and first for cancer mortality in countries with low sociodemographic index [1]. In China, liver cancer is the third leading cause of cancer-related death in 2015 [2]. More than 90% of primary liver cancers are hepatocellular carcinoma (HCC) in the world [3]. Currently, hepatic resection is the main option for the therapy of HCC. However, less than 30% of patients with HCC met the criteria of curative hepatic resection and the over-all 5-year survival rate is still as low as 35-50% due to the high recurrence rate [4, 5]. The availability of treatment options for the patients with intermediate to advanced HCC is very limited. Sorafenib, a molecular targeted drug, has been approved by FDA as the first-line treatment for advanced HCC. However, sorafenib only prolongs about 3 months of survival and the response rate is less than 4% [6, 7]. It is urgent to develop new drugs or strategies against HCC.

Traditional Chinese medicine (TCM) alone or combined with other strategies has been used to treat HCC and shown the clinical benefits including prolonged survival time, improved life quality, reduced adverse reactions, and so on [8, 9]. Cistanche, a kind of TCM, has various biological functions, such as anti-oxidation, anti-inflammation, anti-aging and neuroprotection [10, 11]. Phenylethanoid glycosides have been considered the major active components of Cistanche, which have diverse activities including anti-oxidation, anti-inflammation, hepatoprotection and neuroprotection [12–15]. Our group has reported that *Cistanche tubulosa* phenylethanoid glycosides (CTPG) could induce apoptosis in melanoma B16-F10 cells and inhibited the growth of tumor in mice [16]. In this study, we measured the antitumor effect of CTPG on HCC H22 cells both in vitro and in vivo and investigated its mechanisms. We found that CTPG induced apoptosis in H22 cells through both extrinsic and intrinsic signaling pathways and suppressed the growth of H22 tumor in mice.

## Methods

### Cell line

The mouse H22 hepatocellular carcinoma cells were obtained from the Xinjiang Key Laboratory of Biological Resources and Genetic Engineering, Xinjiang University (Urumqi, Xinjiang, China) and cultured in RPMI 1640 medium (Gibco) supplemented with 100 U/ml penicillin and 100 μg/ml streptomycin, and 10% heat-inactivated fetal bovine serum (Gibco) at 37 °C in a humidified atmosphere of 5% CO_2_.

### MTT assay

CTPG was purchased from Hetian Dichen Biotech Co., Ltd. (Hetian, Xinjiang, China) and the major compounds of CTPG were qualified and quantified by high performance liquid chromatography [16]. Cell viability was evaluated by 3-(4, 5-dimethylthiazol-2-yl)-2, 5-diphenyltetrazolium bromide (MTT) (Sigma, St. Louis, MO, USA) assay. H22 cells were inoculated into 96-well plates at a density of 2 × 10^4^ cells in 100 μl medium per well and cultured at 37 °C. After 24 h, cells were treated with different concentrations of CTPG (0, 100, 200, 300 and 400 μg/ml) or 0.3% DMSO (equal to that in 400 μg/ml CTPG) for 24, 48 and 72 h, respectively. After centrifugation at 1000 rpm for 7 min, supernatant was discarded and 100 μl of MTT solution (5 mg/ml in PBS) was added to each well. The plates were incubated at 37 °C for 4 h and 100 μl DMSO was added to dissolve the formed formazan crystals. The OD_490_ values were detected by a 96-well microplate reader (Bio-Rad Laboratories, CA, USA). The cell viability was calculated according to the formula: Cell viability (%) = (OD_treated_/OD_untreated_) × 100%.

### Detection of apoptosis

H22 cells were treated with different concentrations of CTPG (0, 100, 200, 300 and 400 μg/ml) or 0.3% DMSO for 24 h, and then stained with Annexin V-FITC/Propidium iodide (PI) Apoptosis Detection Kit (YEASEN, China) according to the manufacturer’s instructions. Samples were analyzed by flow cytometry (BD FACSCalibur, USA).

### Detection of mitochondrial membrane potential

H22 cells were treated with different concentrations of CTPG (0, 200 and 400 μg/ml) for 24 h, and then stained with the membrane-permeable JC-1 dye (Beyotime,China) for 20 min at 37 °C. After washing twice with JC-1 buffer, samples were resuspended with 300 μl of JC-1 buffer and analyzed by flow cytometry (BD FACSCalibur, USA).

### Analysis of cell cycle

H22 cells were inoculated in 60 mm culture dishes and treated with different concentrations of CTPG (0, 100, 200, 300 and 400 μg/ml) or 0.3% DMSO for 24 h. All cells were collected and washed twice with PBS. Cells were fixed in 70% ice-cold ethanol at − 20 °C for 2 h and washed twice with PBS, then re-suspended in 300 μl Propidium iodide/RNase staining buffer (BD Biosciences). After 10 min at room temperature, samples were collected by flow cytometry (BD FACSCalibur, USA) and cell cycle distribution was analyzed with the ModFit LT 3.0 software.

### Hoechst 33,258 staining

The morphological changes of H22 cell nuclei were analyzed by membrane-permeable DNA-binding dye Hoechst 33,258 staining. H22 cells were seeded in 6-well plate at the concentration of 1 × 10^5^ cells/well in 2 ml medium. After 60%~ 70% confluence, the cells were treated with CTPG (0, 100, 200, 300 and 400 μg/ml) for 24 h. The cells were collected and fixed with 4% ice-cold Paraformaldehyde at 4 °C for 10 min. After washing with PBS, cells were stained with Hoechst 33,258 (Beyotime, China) at 4 °C for 10 min. Samples were observed by inverted fluorescence microscope (Nikon Eclipse Ti-E, Japan).

### Western blot

Anti-caspase-3, anti-cleaved caspase-3, Anti-Bcl-2 and anti-Bax were purchased from Beyotime Biotech Co., Ltd. (Shanghai, China). Anti-caspase-7, anti-cleaved-caspase-7, anti-caspase-8, anti-cleaved-caspase-8, anti-caspase-9, anti-cleaved-caspase-9, anti-PARP, anti-cleaved PARP, anti-mouse IgG-HRP and anti-rabbit IgG-HRP were purchased from Cell Signaling Technology. Anti-β-actin was purchased from Beijing ComWin Biotech Co., Ltd. (Beijing, China).

H22 cells were treated with different concentrations of CTPG (0, 100, 200, 300 and 400 μg/ml) or 0.3% DMSO for 24 h. Cells were collected and lysed with the Cell Lysis Solution RIPA (Beijing ComWin Biotech Co., Ltd) for 30 min on ice. Samples were spun down (12,000 g for 15 min at 4 °C) to collect the supernatants and protein concentrations were measured by BCA Kit (Thermo Fisher Scientific, USA). Equal amount of protein in each sample was isolated by 12% SDS-PAGE and transferred to PVDF membranes (Biosharp, China). After blocking with TBST buffer contained 5% nonfat milk, membranes were incubated with corresponding primary antibodies and secondary antibodies conjugated to horseradish peroxidase (HRP), respectively. After washing with TBST, the target proteins were detected by ECL assay kit (Beyotime, China).

### Animals and ethics statement

6-8 weeks old male Kunming mice were purchased from Animal Laboratory Center, Xinjiang Medical University (Urumqi, Xinjiang, China). Mice were kept in a standard temperature-controlled, light-cycled animal facility of Xinjiang University. All animal studies were carried out according to the guidelines of the Animal Care and Use Committee of Xinjiang University. The protocol was approved by the Committee on the Ethics of Animal Experiments of Xinjiang Key Laboratory of Biological Resources and Genetic Engineering (BRGE-AE001), Xinjiang University.

### Tumor mouse study

For induction of tumor mouse model, male Kunming mice were subcutaneously injected with 1 × 10^6^ H22 cells in 100 μl PBS into the right flank. After 3 days, mice were randomly divided into 3 groups (7 mice/group). Control group was injected with 0.1 ml DMSO subcutaneously around tumor. CTPG-200 and CTPG-400 groups were subcutaneously injected with 200 or 400 mg/kg CTPG in 0.1 ml DMSO around tumor. Mice were treated every 2 days for up to 21 days. Tumor sizes were measured using calipers up to 25 days and tumor volume was calculated according to the formula: tumor volume (mm^3^) = (length×width^2^)/2. After 25 days, survival of tumor mice was monitored every day until the end of this study.

### Statistical analysis

Statistical significance was calculated by one-way analysis of variance among the treatment and control groups. All data were expressed as the mean ± standard deviation (S.D.). *p* < 0.05 was considered statistically significant.

## Results

### CTPG reduced the viability of H22 cells in vitro

**Fig. 1:**
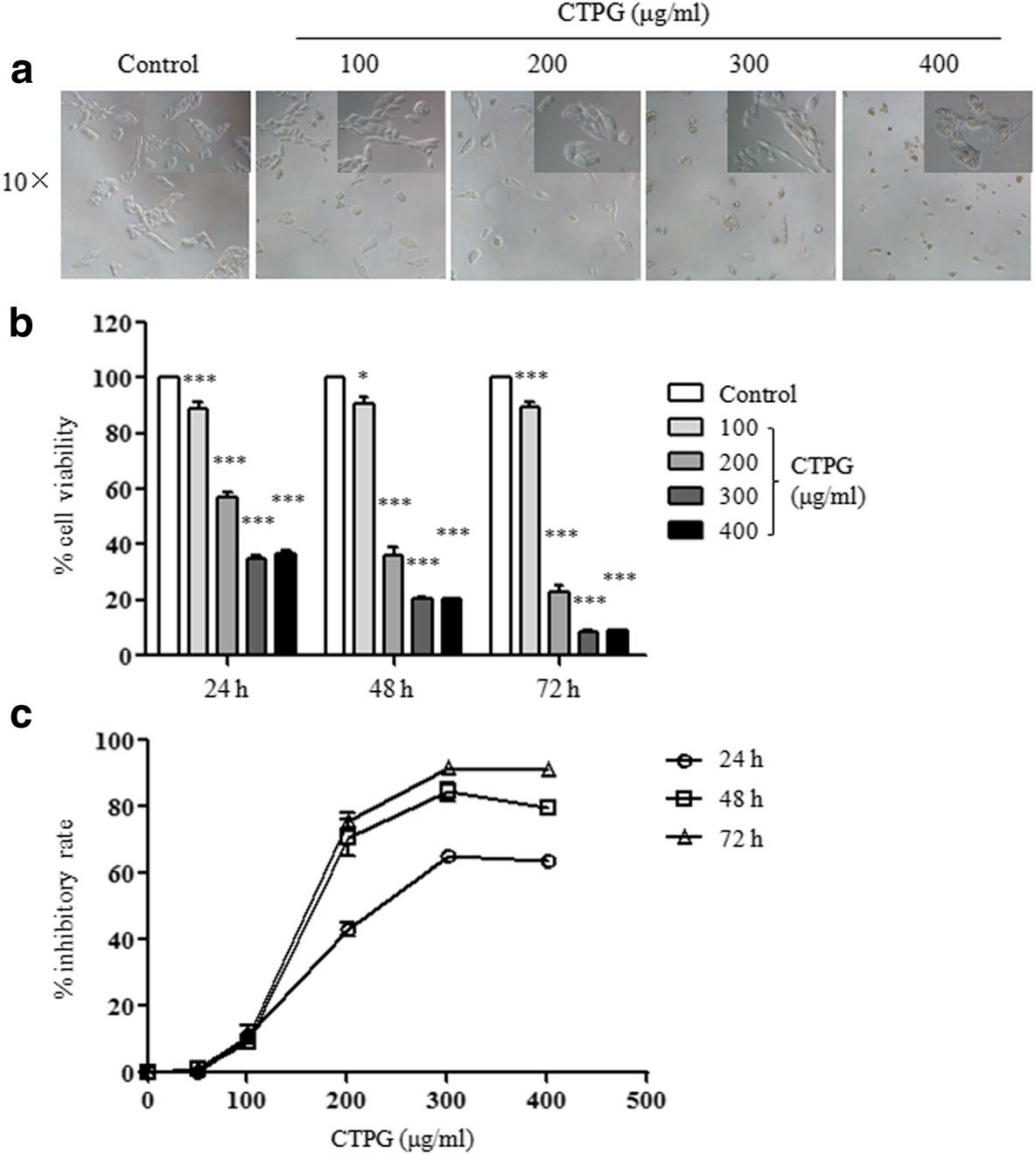
The effect of CTPG on the growth of H22 cells. H22 cells were treated with different concentrations of CTPG. (**a**) After 24 h, the morphology of H22 cells was observed by inverted microscope. Inserted panels: 20 folds of magnification. (**b**) After 24, 48 and 72 h, cell viability was detected by MTT assay. (**c**) The inhibitory rate of CTPG. * *p* < 0.05; *** *p* < 0.001 compared to control

In order to explore antitumor effect of CTPG on HCC, H22 cells were treated with different concentrations of CTPG (0, 100, 200, 300 and 400 μg/ml) in vitro. After 24 h, the morphology of H22 cells was observed using inverted microscope. We found that the morphology of H22 cells was dramatically changed by CTPG treatment. With increasing CTPG concentration, cells became small and round and cell number was also greatly reduced (Fig. 1a). MTT assay was used to analyze the viability of H22 cells after CTPG treatment for 24, 48 and 72 h, respectively. CTPG significantly reduced H22 cell viability in a dose-dependent and time-dependent manner (Fig. 1b). CTPG at 300 μg/ml arrived at the best inhibitory rate (Fig. 1c). The values of IC_50_ of CTPG for H22 cells are 236 μg/ml at 24 h and 169.8 μg/ml at 48 h.

### CTPG induced apoptosis in H22 cells

**Fig. 2:**
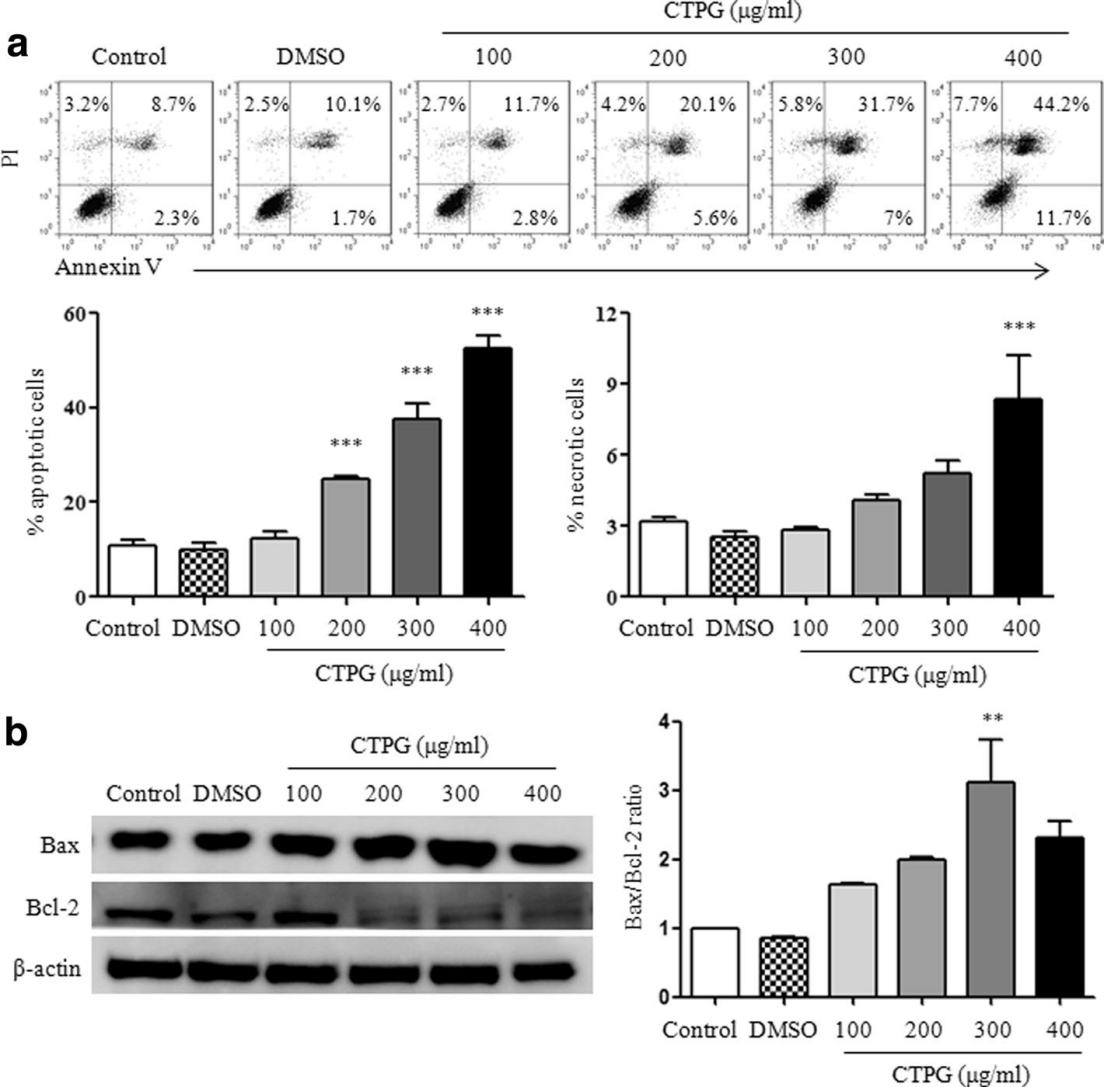
CTPG induced apoptosis in H22 cells. Cells were treated with different concentrations of CTPG for 24 h. (**a**) The apoptotic and necrotic H22 cells were detected by flow cytometry. The upper panel showed the individual dot plots and the lower panel showed the summary data. (**b**) Total protein was isolated to analyze the expressions of Bax and Bcl-2 by Western blot. ** *p* < 0.01; *** *p* < 0.001 compared to control

To investigate whether the decreased viability of H22 cells is mediated by the induction of apoptosis, H22 cells were treated with different concentrations of CTPG (0, 100, 200, 300 and 400 μg/ml) for 24 h and stained with PI and Annexin V. The flow cytometry results showed that CTPG significantly induced apoptosis of H22 cells (including early and late apoptosis) in a dose-dependent manner (Fig. 2a). Although the high dose of CTPG also significantly increased necrosis of H22 cells, necrosis plays a minor role in the inhibition of H22 cell growth due to its lower proportion (8.3%) compared to that of apoptosis (52.6%). Further, total proteins of H22 cells were isolated after CTPG treatment and the expressions of anti-apoptotic B cell lymphoma 2 (Bcl-2) and pro-apoptotic BCL-2-associated X protein (Bax) were detected by Western blot. Grayscale scanning data showed that the expression levels of Bax and Bcl-2 were increased and decreased, respectively. The Bax/Bcl-2 ratio was significantly increased (Fig. 2b). These results suggest that CTPG induces apoptosis in H22 cells.

### CTPG induces chromosomal condensation and cell cycle arrest in H22 cells

**Fig. 3:**
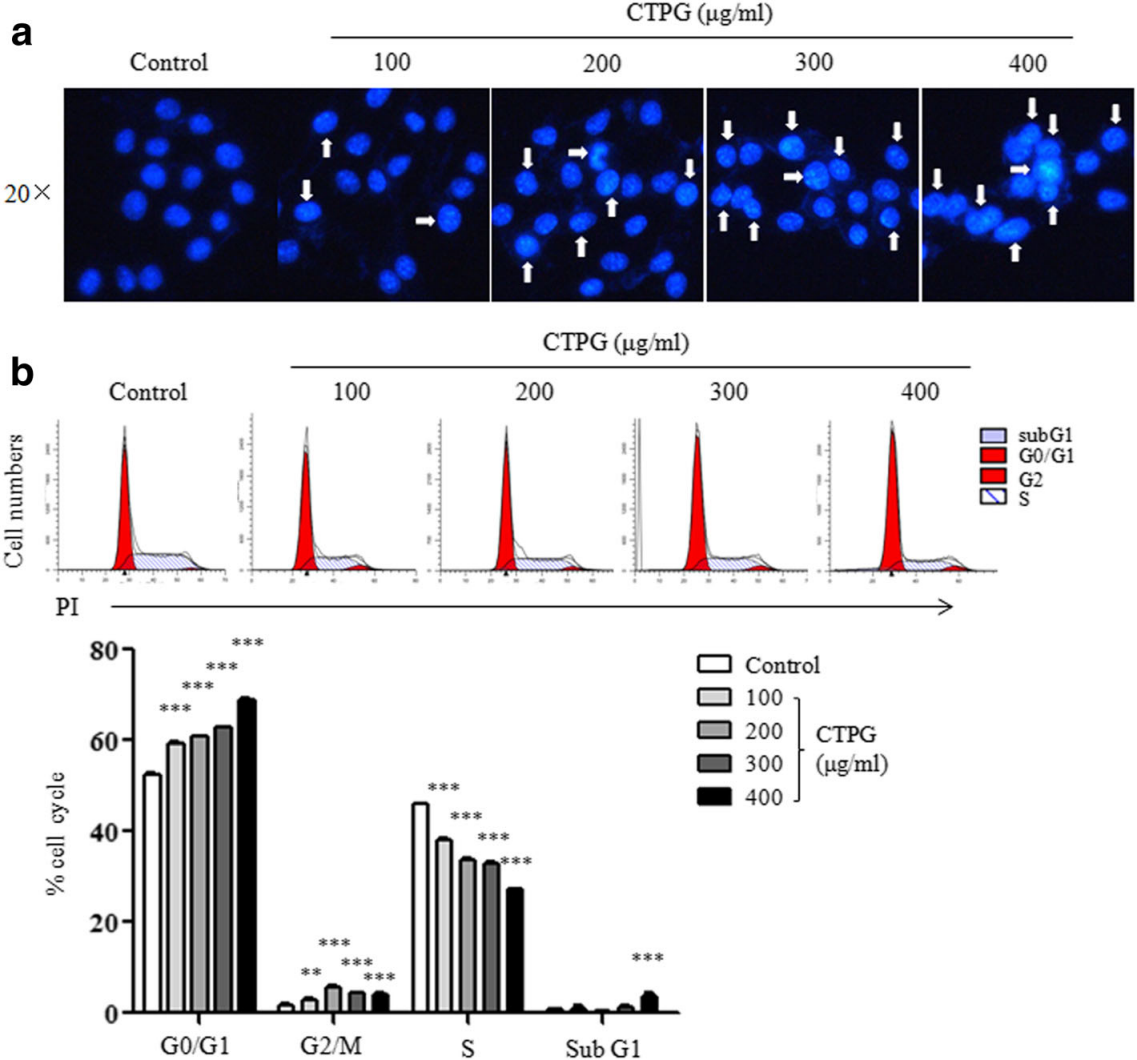
CTPG induced chromosomal condensation and cell cycle arrest in H22 cells. H22 cells were treated with different concentrations of CTPG for 24 h. (**a**) Cells were stained with Hoechst 33,342 and observed by inverted fluorescent microscopy. The arrows indicated the chromosomal condensation or fragmentation. (**b**) Cell cycle distribution in H22 cells was analyzed by flow cytometry. ** *p* < 0.01; *** *p* < 0.001 compared to control

It has reported that DNA damage and cell cycle arrest induced by drugs can inhibit tumor cell growth and cause apoptosis in tumor cells [17, 18]. To detect the morphology of nuclei in H22 cells after CTPG treatment for 24 h, H22 cells were stained by Hoechst 33,342 and observed using inverted fluorescent microscopy. CTPG-treated cells showed a dose-dependent increase of brightly condensed chromatin of nuclei, while the untreated cells showed the homogeneously stained nuclei (Fig. 3a). Cell cycle distribution in H22 cells was further analyzed by PI staining after CTPG treatment for 24 h. As shown in Fig. 3b, CTPG treatment significantly increased the proportion of G0/G1- and G2/M-phase cells and significantly decreased the proportion of S-phase cells, suggesting that CTPG induced G0/G1 and G2/M-phase arrest in H22 cells. The high dose of CTPG also significantly increased the proportion of sub G1 cells.

### CTPG decreased mitochondrial membrane potential and increased the release of cytochrome c

**Fig. 4:**
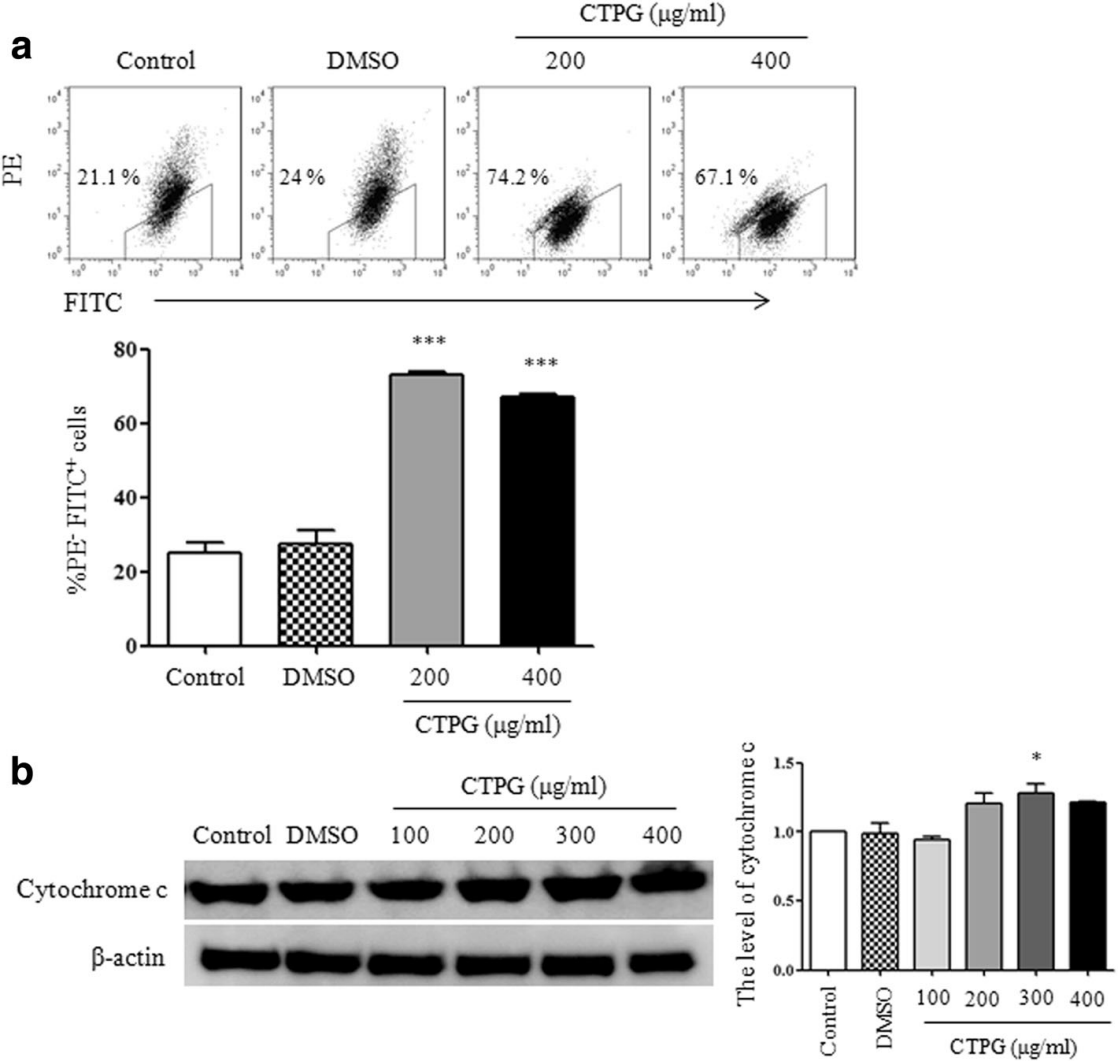
The reduction of Δψm and release of cytochrome c. H22 cells were treated with different concentrations of CTPG for 24 h. (**a**) Cells were stained with JC-1 dye and analyzed by flow cytometry. The individual dot plots show the changes of JC-1 fluorescence. The summary data are shown in the lower panel. (**b**) Total protein was isolated to detect the release of cytochrome c by Western blot. * *p* < 0.05; *** *p* < 0.001 compared to control

Mitochondrial-dependent pathway plays an important role in the induction of apoptosis [19, 20]. The changes of mitochondrial membrane potential (Δ*ψ*m) can be monitored by JC-1 staining due to JC-1 aggregate (red fluorescence) can disintegrate into monomer (green fluorescence) with the reduction of Δ*ψ*m [21]. After CTPG treatment for 24 h, H22 cells were stained by JC-1 dye. The flow cytometry data showed that the red fluorescence in FL-2 channel and green fluorescence in FL-1 channel were significantly decreased and increased upon CTPG treatment. The proportion of PE^−^FITC^+^ cells were significantly increased (Fig. 4a), suggesting that CTPG reduced the Δ*ψ*m in H22 cells. This is consistent with the increased Bax/Bcl-2 ratio. Consequently, we observed the release of cytochrome c was significantly increased upon CTPG treatment (Fig. 4b). These results indicated that CTPG might partially induce apoptosis in H22 cells via mitochondrial-dependent (intrinsic) pathway.

### CTPG activated caspase pathway and prevented DNA repair

**Fig. 5:**
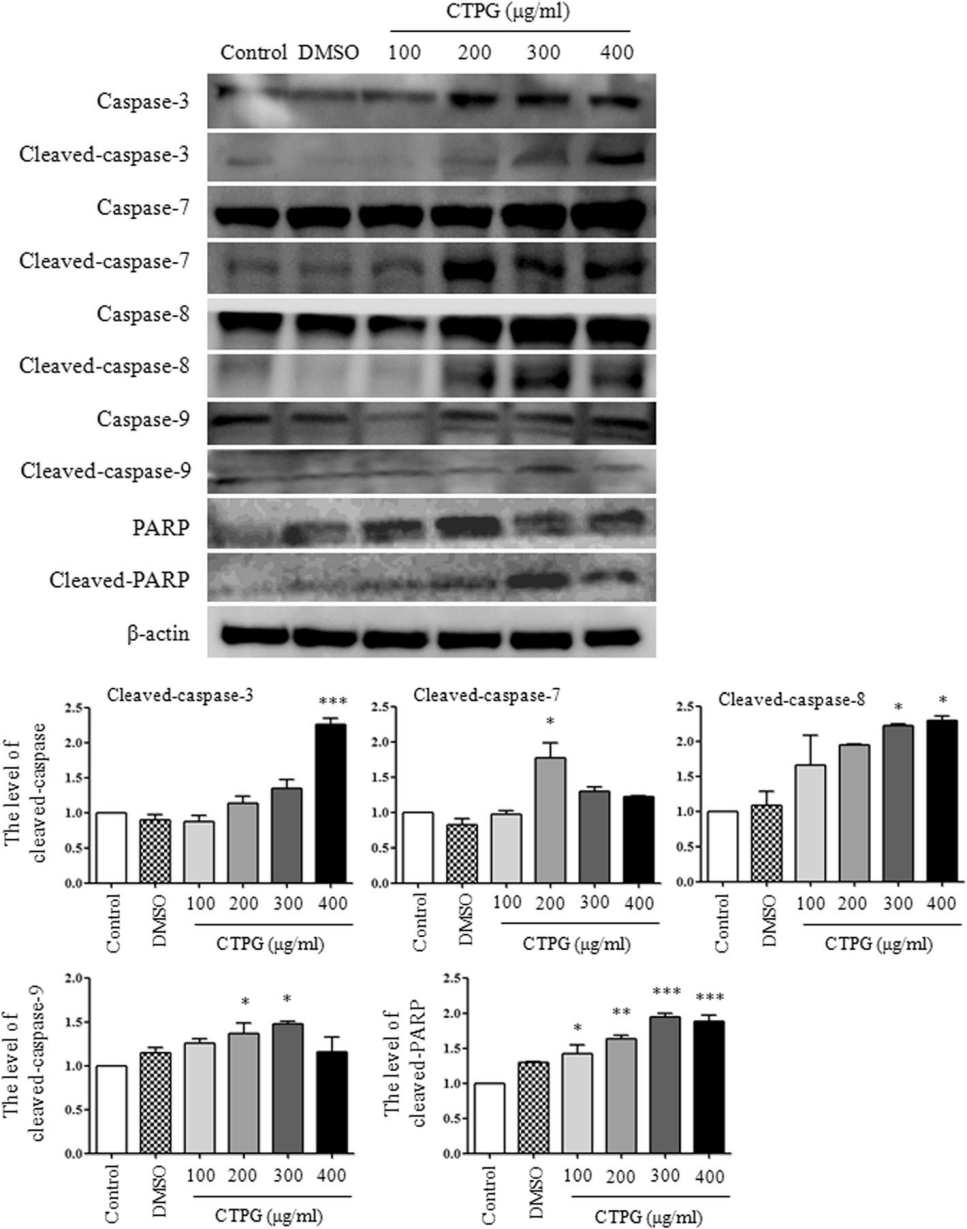
The levels of cleaved-caspases and cleaved-PARP. H22 cells were treated with different concentrations of CTPG for 24 h. Total protein was isolated to detect the levels of cleaved-caspases and cleaved-PARP by Western blot

Next, the activation of caspase induced by CTPG via both extrinsic and intrinsic signaling pathways was analyzed. After CTPG treatment for 24 h, total proteins were isolated from H22 cells and the levels of pro- and cleaved-caspases were detected by Western blot. Compared with the untreated or the DMSO control, CTPG treatment significantly up-regulated not only the level of cleaved caspase-8 (extrinsic pathway) but also the level of cleaved caspase-9 (intrinsic pathway) (Fig. 5). Sequentially, activated caspase-8 and -9 cleaved the downstream pro-caspase-3 and -7 that was observed in Fig. 5. Activated caspase-3 cleaved the DNA repair enzyme of poly (ADP-ribose) polymerase (PARP) to prevent DNA repair and accumulate DNA damage as observed in Fig. 3a. These results indicated that CTPG induced apoptosis in H22 cells through both extrinsic and intrinsic signaling pathways.

### CTPG suppresses the growth of H22 HCC in vivo and improves the survival rate of tumor mice

**Fig. 6:**
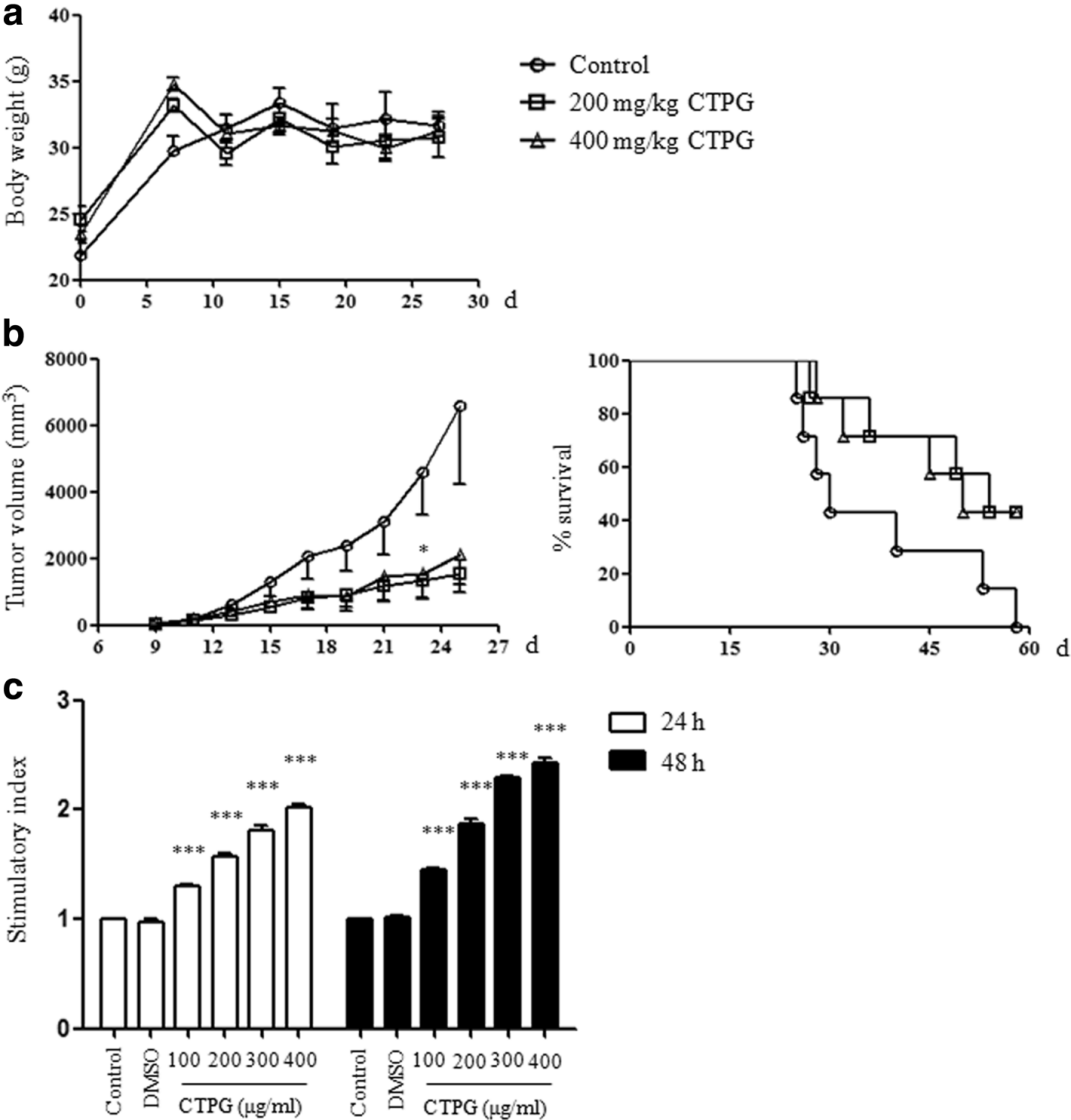
CTPG suppressed tumor growth in vivo. Tumor mouse model was established by injection of H22 cells. After 3 days, tumor mice (7 mice/group) were treated with or without CTPG. Body weight of mice (**a**), tumor sizes and survival rate (**b**) were monitored at the indicated time points. (**c**) Splenocytes were isolated from Kunming mice and treated with different concentrations of CTPG for 24 and 48 h. The proliferation of splenocytes was analyzed by MTT. * *p* < 0.05; *** *p* < 0.001 compared to control

Finally, the antitumor effect of CTPG on HCC was evaluated in tumor mouse model, which was established by subcutaneous injection of H22 cells. After 3 days of H22 cell injection, tumor mice were treated with CTPG for 8 times. The body weight of mice and tumor sizes were monitored at indicated time points. As shown in Fig. 6a, the body weight of mice in each group has no significant difference, suggesting that the selected doses of CTPG have no obvious side effect. Interestingly, the tumor growth in mice treated with both 200 mg/kg and 400 mg/kg of CTPG was significantly inhibited (Fig. 6b). Moreover, the two doses of CTPG treatment greatly improved the survival of tumor mice (3/7, 3/7) compared with control group (0/7) at the end of the experiment (Fig. 6b). We also found that CTPG significantly enhanced the proliferation of splenocytes isolated from male Kunming mice in a dose-dependent manner (Fig. 6c), suggesting that CTPG has immunostimulatory effect.

## Discussion

TCM has been used to treat various diseases including cancers for a long history. It has been reported that TCM can induce apoptosis in different types of tumor cells through both extrinsic (death receptor-mediated) and intrinsic (mitochondria-dependent) signaling pathways to exert antitumor effects [22–25]. The two pathways can activate caspase-8 and -9, respectively [24, 26]. Here, we found that CTPG significantly suppressed H22 cell growth through induction of apoptosis and cell cycle arrest. The levels of cleaved caspase-8 and -9 were significantly up-regulated by CTPG treatment, suggesting that both extrinsic and intrinsic signaling pathways were involved in the induction of apoptosis. Our previous study showed that CTPG induced apoptosis in melanoma B16-F10 cells by mitochondria-dependent pathway that increased the level of cleaved caspase-9 but not caspase-8 [16]. CTPG might activate distinct signaling pathways in different types of tumor cells.

Mitochondrial membrane integrity is tightly regulated by the members of the BCL-2 protein family including Bax and Bcl-2 [27, 28]. The ratio of Bax to Bcl-2 plays a critical role in mitochondria-dependent apoptosis pathway [29]. In H22 cells treated by CTPG, Bax/Bcl-2 ratio was significantly up-regulated, which might cause the reduction of Δ*ψ*m and the release of cytochrome c observed in this study. Consequently, pro-caspase-9 was cleaved and activated. Finally, the initiators of active caspase-8 and -9 activated the executioner of caspase-3 to cleave PARP to prevent DNA repair. Taken together, these results suggested that CTPG induced apoptosis in H22 cells through both extrinsic and intrinsic signaling pathways.

In tumor mouse model, CTPG significantly suppressed the growth of H22 HCC and greatly improved the survival of tumor mice. Interestingly, CTPG dose-dependently promoted the proliferation of splenocytes from Kunming mice, which is consistent with our previous study [16]. These results suggested that CTPG might suppress the growth of H22 HCC in mice through both direct antitumor effect and indirect immune enhancement.

## Conclusions

CTPG suppressed the growth of H22 cells both in vitro and in vivo and induced apoptosis in H22 cells through both extrinsic and intrinsic signaling pathways. These data indicated that CTPG might be a potential candidate for the treatment of HCC.

## Abbreviations

Bax: BCL-2-associated X protein
Bcl-2: B cell lymphoma 2
CTPG: *Cistanche tubulosa* phenylethanoid glycosides
HCC: hepatocellular carcinoma
HRP: horseradish peroxidase
MTT: 3-(4, 5-dimethylthiazol-2-yl)-2, 5-diphenyltetrazolium bromide
PARP: DNA repair enzyme of poly (ADP-ribose) polymerase
TCM: traditional Chinese medicine
Δψm: mitochondrial membrane potential
